# Combined Catalysis: A Powerful Strategy for Engineering Multifunctional Sustainable Lignin-Based Materials

**DOI:** 10.1021/acsnano.3c00436

**Published:** 2023-04-04

**Authors:** Samson Afewerki, Ulrica Edlund

**Affiliations:** † Fibre and Polymer Technology, KTH Royal Institute of Technology, SE 100 44 Stockholm, Sweden

**Keywords:** combined catalysis, lignin, antimicrobial, self-healing, adhesive, green chemistry, valorization, sustainable material, organic synthesis, organohydrogel

## Abstract

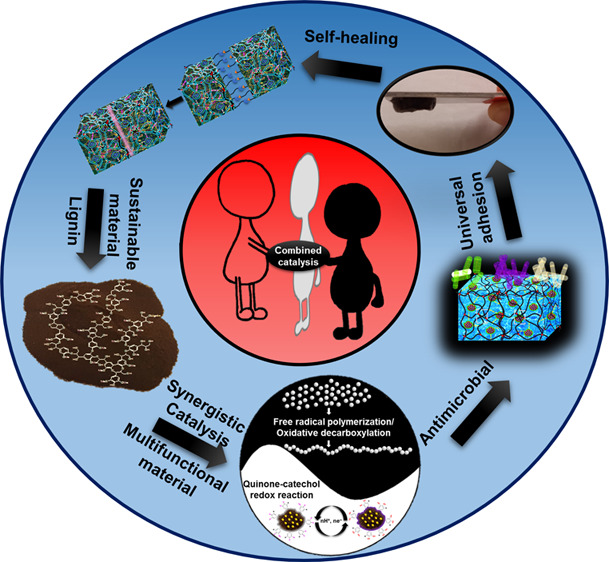

The production and engineering of sustainable materials through green chemistry will have a major role in our mission of transitioning to a more sustainable society. Here, combined catalysis, which is the integration of two or more catalytic cycles or activation modes, provides innovative chemical reactions and material properties efficiently, whereas the single catalytic cycle or activation mode alone fails in promoting a successful reaction. Polyphenolic lignin with its distinctive structural functions acts as an important template to create materials with versatile properties, such as being tough, antimicrobial, self-healing, adhesive, and environmentally adaptable. Sustainable lignin-based materials are generated by merging the catalytic cycle of the quinone–catechol redox reaction with free radical polymerization or oxidative decarboxylation reaction, which explores a wide range of metallic nanoparticles and metal ions as the catalysts. In this review, we present the recent work on engineering lignin-based multifunctional materials devised through combined catalysis. Despite the fruitful employment of this concept to material design and the fact that engineering has provided multifaceted materials able to solve a broad spectrum of challenges, we envision further exploration and expansion of this important concept in material science beyond the catalytic processes mentioned above. This could be accomplished by taking inspiration from organic synthesis where this concept has been successfully developed and implemented.

## 1 Introduction

**Figure 1:**
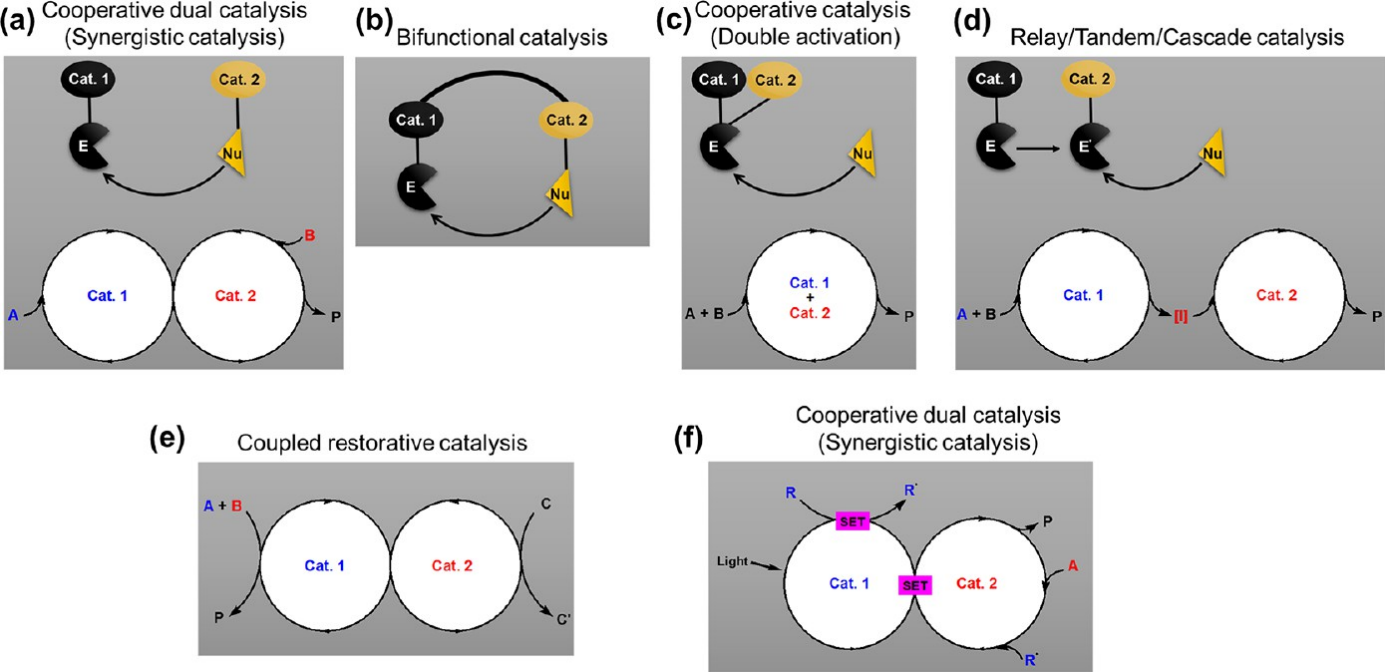
Examples of various classifications of catalytic systems using two catalysts and multicatalytic systems with their respective catalytic cycles. (a) Cooperative catalysis or synergistic catalysis.^6^ (b) Bifunctional catalysis.^7^ (c) Cooperative catalysis (double activation).^8,9^ (d) Relay/tandem/cascade catalysis.^10−13^ (e) Coupled restorative catalysis.^14,15^ (f) Cooperative catalysis or synergistic catalysis involving SET through photoredox catalysis.^16−19^ Cat. = catalyst; E = electrophile; Nu = nucleophile; A = substrate 1; B = substrate 2; C = oxidant; C′= reductant; R = substrate; R^•^ = a radical compound; *P* = product; SET = single-electron transfer.

The use of catalysis for various chemical transformations in organic synthesis has been demonstrated to be a highly powerful and efficient approach.^1^ This approach allows chemists to construct and build highly complex molecular structures^2^ and chemicals sustainably and efficiently.^3^ The great impact of this research field has recently led to the Nobel Prize in Chemistry (2021) for the development of asymmetric organocatalysis awarded to Benjamin List and David MacMillan.^4^ In this review, we will start by providing some examples of catalytic systems involving two catalysts and some multicatalytic systems and their classifications used in organic synthesis in the vision to inspire innovations in material science. When two catalysts are used to promote a chemical transformation, the concept of combined catalysis can be classified on the basis of the catalyst’s activations of substrates forming the electrophile (a chemical species that accepts an electron pair and forms a bond with a nucleophile) and/or nucleophile (a chemical species that forms a bond by donating an electron pair).^5^ This powerful chemical concept allows the coupling of these two reactive species and the formation of an innovative chemical bond that otherwise is not attainable by using one of the catalysts or catalytic systems alone. In cooperative dual catalysis or synergistic catalysis, the two catalysts independently and without interferences activate the two reactive species, an electrophile and a nucleophile, through two distinct catalytic cycles to lead to a new chemical bond (Figure 1a).^6^ Nevertheless, when the nucleophile and electrophile are instead activated by one catalyst containing two catalytic sites, the catalytic process is called bifunctional catalysis (Figure 1b).^7^ In cooperative catalysis or double activation catalysis, the chemical process proceeds in one single catalytic cycle, and the two catalysts work cooperatively to activate one of the substrates, for instance, by generating a reactive electrophile activated by both catalysts (Figure 1c).^8,9^ In a relay,^10^ that is, tandem or cascade catalysis, the two catalysts activate the same substrates but in a sequential manner, where one of the catalysts initially activates one substrate to generate an intermediate [I] that is subsequently activated by the second catalyst (Figure 1d).^11−13^ The coupled restorative catalysis is a catalytic redox reaction where it allows the use of a terminal oxidant or redox equivalent that could not be used otherwise, and during the process, the catalyst is reoxidized by another catalyst while the product is simultaneously generated (Figure 1e).^14,15^ Another catalytic strategy is the activation of a single electron in a single-electron transfer (SET) pathway through photoredox catalysis, where metal complexes and organic dyes convert visible light to chemical energy to generate reactive intermediates. Subsequently, the generated intermediates can further be merged into the second catalytic cycle and, through synergistic catalysis, lead to the formation of a new chemical bond (Figure 1f).^16−19^ In this review, we will also discuss some examples where one catalyst will be involved in two intertwined catalytic cycles, which triggers innovative activation modes and material properties.^19^ All the above-highlighted catalytic systems have been highly recognized and respected in organic synthesis.^20^ Some of them are widely observed in biological systems,^21,22^ which allows the formation of specific chemical bonds and molecules.^23^

**Figure 2:**
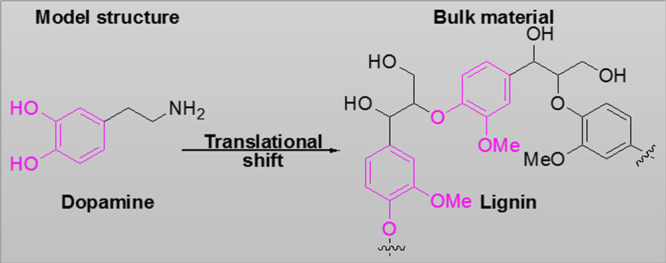
A strategic translational shift from the fundamental chemical understanding of a model structure, such as dopamine, into a translational shift to structurally complex bulk materials, such as lignin.

Despite the powerful catalytic platforms these catalytic systems display, their transformations into material science or polymer chemistry applications have been very limited and far from the level achieved within the field of organic synthesis. Hence, we envisioned that the transformation of this concept into material design and engineering applications could expand the organic chemistry research area and at the same time promote material scientists to design tailor-made materials and create innovations efficiently and sustainably.^24^ As chemists have taken lead and inspiration from nature, the material chemistry research field could gain some inspiration from the field of organic synthesis. The translational shift from the conventional field of catalytic reactions in small molecular systems into bulk materials and larger systems, such as polymers, could be smoothly transitioned by using model reactions to decrease and simplify the complexity to gain a more profound understanding, and then that information could be further applied to material design and other important applications (Figure 2). However, there are a couple of reports on the use of combined catalysis in material science, as we will highlight in this review, but they are very limited, and there is still room for further expansion of this significant research topic. The hope is that this review will promote innovative combined catalytic systems in material science and also create a fundamental understanding of the mechanism of action at a deeper level.

To meet the increasing demand for sustainable materials, we need approaches that promote the creation of tailor-made materials that have multifaceted functions and properties able to solve various challenges simultaneously without interference. Examples of tailor-made materials are, for instance, materials engineered to become thermoelectric,^25−27^ conductive,^28,29^ piezoelectric,^30,31^ ferroelectric,^32^ adsorptive,^33−35^ reversibly disintegrated, and chemically recyclable.^36−40^ These types of materials will play a major role in a sustainable society, in particular, if these materials are engineered through green chemistry.^41,42^ For instance, multifunctional materials that are durable, self-healing, antimicrobial, and adhesive would play a significant role in application and provide a plethora of solutions.^43^ We truly believe that materials with self-healing ability have the potential to revolutionize the application of materials in our daily life. This characteristic could lead to a more sustainable and robust material that could prevent any critical damage to the material by immediately recovering to its original structure and properties after sudden damage.^44^ Furthermore, the global health threat from the prevalence of microbial infections is a major global challenge leading to increased healthcare costs and antibiotic resistance in pathogenic microorganisms; if innovations are not made to solve this increasing challenge, it will unquestionably lead to a catastrophic event.^45−47^ From this perspective, multifunctional materials with antimicrobial properties would provide a complementary solution to the overuse of antibiotics and hopefully minimize or in some applications avoid the use of antibiotics.^43,48^ Additionally, the adhesive ability of a multifunctional material would further broaden its applications and features by promoting good integration with the desired surface, thereby forming a strong barrier and staying intact.^49,50^

## 2 Lignin: A Versatile Material

**Figure 3:**
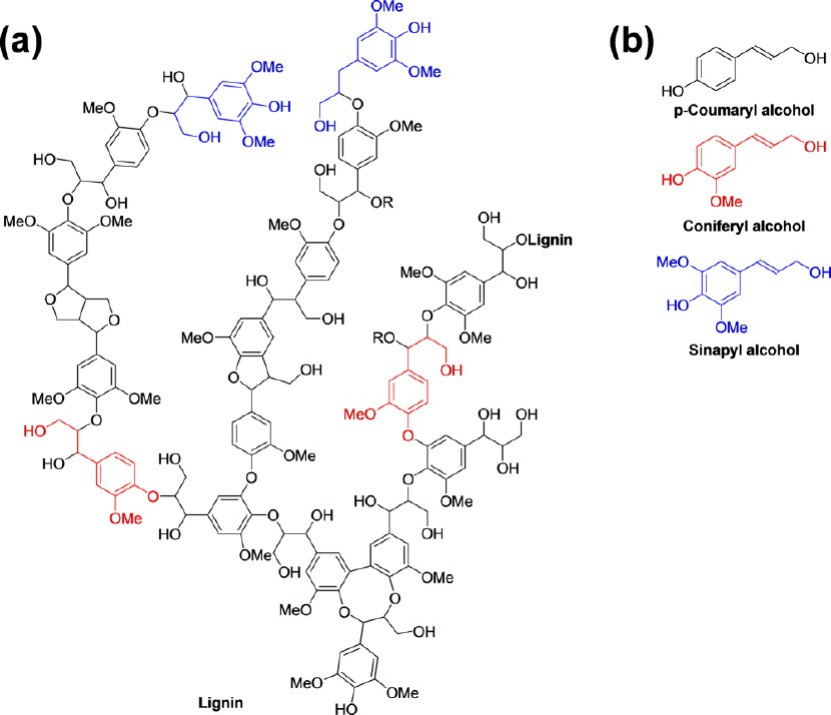
(a) Representative structural motif of softwood lignin^74^ and (b) the main precursors.

Lignin is one of the most abundant biopolymers on earth.^51^ It constitutes about a third of the mass of all wood biomass and consists of a highly complex polyaromatic structure linked with primary structure phenylpropanol precursors, such as *p*-coumaryl-, coniferyl-, and sinapyl alcohol (Figure 3).^52,53^ It is noteworthy that several lignin types can be obtained on the basis of the processing approach used to extract the lignin, such as soda lignin,^54^ Kraft lignin,^53,55^ hydrolyzed lignin,^56−58^ organosolv lignin,^59,60^ and lignosulfonates.^61,62^ Moreover, there are a wide range of lignin sources available, such as wood (e.g., conifer, deciduous tree, etc.) and other terrestrial plants (e.g., wheat straw, rye straw, canola, alfalfa, jute, hemp, coir, kenaf, etc.).^63^ All these various extraction processing approaches and lignin sources provide lignin with different physical and chemical behaviors and with varying lignin structures (linear/branched), characteristics, functional groups, and performances.^64^ Hence, in the context of this review, it would be very interesting to evaluate the various lignin types and lignin extracted from various sources to assess their catalytic performance. Lignin obtained as a byproduct from wood pulp production has only low added-value applications and is often incinerated. Thus, the development of facile and versatile technologies for the valorization^65^ of a large amount of lignin isolated during the production of pulp would generate great profit and complement fossil-based products.^66^ Particularly, the invention of technologies that allow the use of raw lignin without any further complicated processing or chemistry would facilitate commercialization and scalability. Lignin has further demonstrated features such as antibacterial,^67,68^ antioxidant,^69^ and ultraviolet shielding properties.^70^ Despite that, there are already many materials generated from lignin, such as carbon fibers, plastics, polymeric foams, and membranes,^71,72^ and though important advancements have been made, there is still room for further development and innovation. Tailor-made and multifunctional sustainable lignin-based materials could serve in various environmental, biomedical, and tissue engineering applications, such as drug delivery vehicles, antimicrobial patches, bioink for three-dimensional (3D) printing, flexible electronics, medical devices, coating materials in packaging, etc.^73^

**Figure 4:**
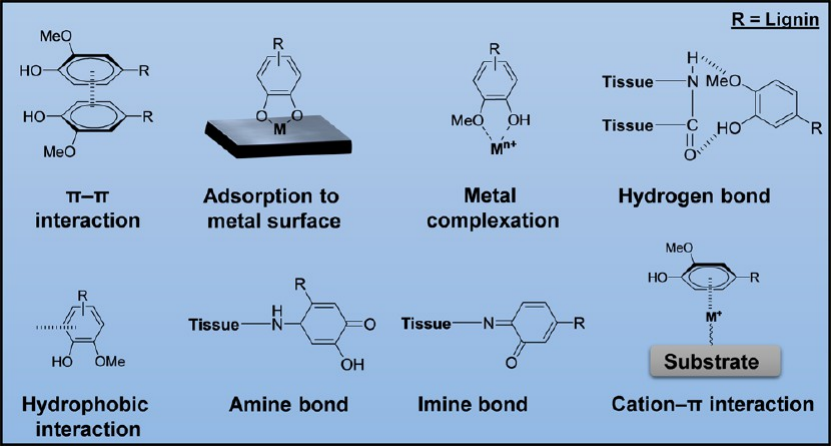
Possible interaction potentials of lignin and its precursors, such as π–π interactions with benzene groups, adsorption to metal surfaces, metal complexation, hydrogen bonds, hydrophobic interactions, formation of amine and imine bonds, and cation−π interactions.

The inimitable structure of lignin with an abundance of vital phenolic groups, such as derivatives of catechol and pyrogallol groups, makes it highly desirable for engineering multifunctional biomaterials. These structural moieties have been shown to promote a wide range of interaction possibilities and have also been widely observed in nature and biological systems.^75,76^ The various interactions are π–π interactions with benzene groups, adsorption to metal surfaces, metal complexation, the formation of hydrogen bonds through methoxy or hydroxyl groups, hydrophobic interactions with the benzene groups, amine and imine bonds formations (e.g., with tissues), and cation−π interactions between metal ions and the benzene groups (Figure 4).^77,78^

**Figure 5:**
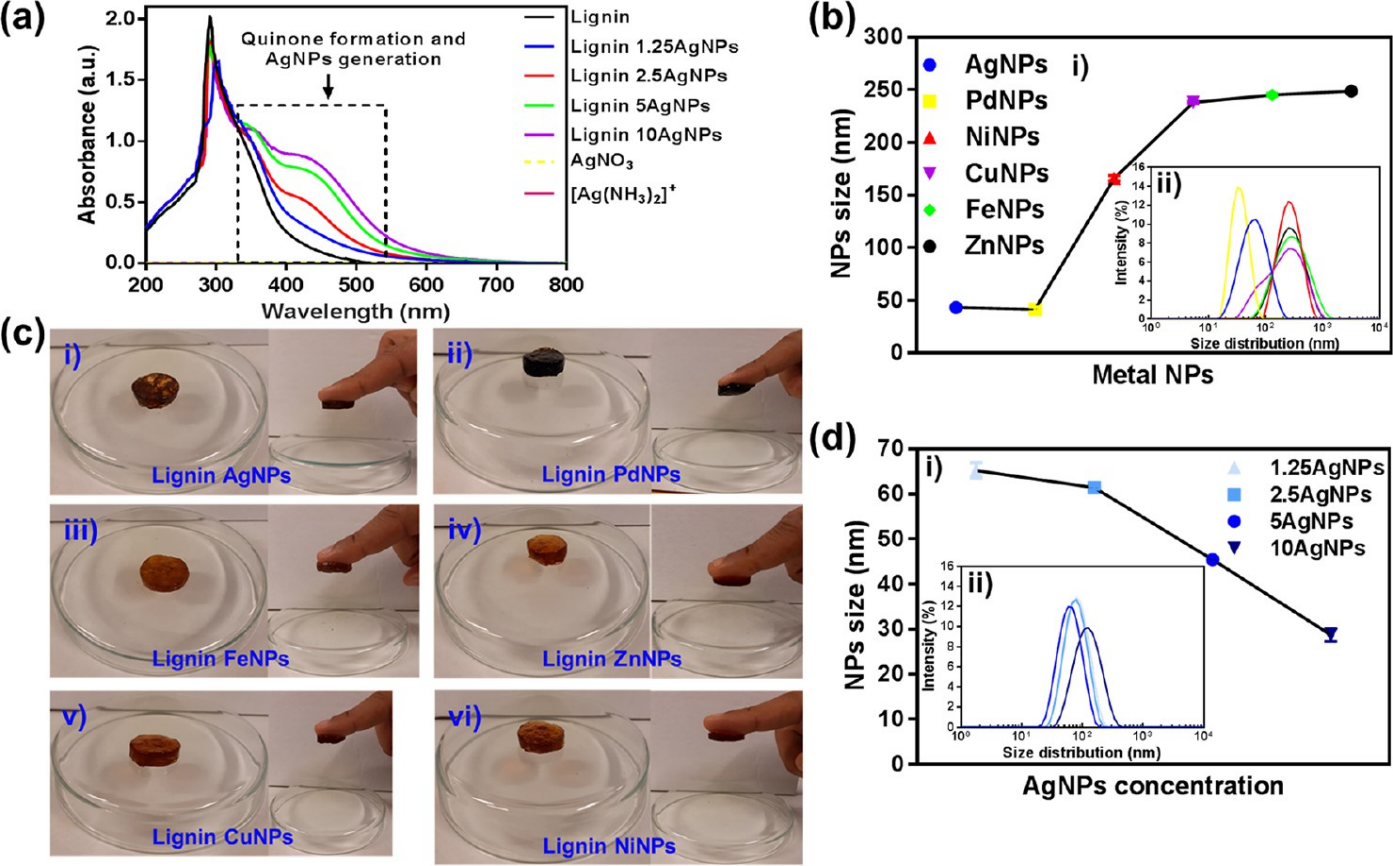
(a) UV–vis spectrum demonstrating the quinone formation and the presence of the lignin AgNPs with various silver concentrations. (b) Figure showing various lignin metal NPs and their NPs sizes. (c) Photos demonstrating the adhesive ability of various multifunctional hydrogels engineered through various lignin metal NPs. (d) Figure showing the sizes of lignin AgNPs and the impact on the NPs sizes from using various concentrations of silver. Reproduced with permission from ref (83). Copyright 2020, American Chemical Society.

Lignin, such as lignosulfonate and Kraft lignin, has been used as a green reducing agent and stabilizing agent to generate various metal-based nanoparticles (NPs)^79,80^ by oxidizing the catechol and pyrogallol derivative moieties within lignin to the corresponding quinone/hydroquinone groups (Figure 5a).^81,82^ Various lignin metal NPs, for example, with Ag, Pd, Cu, Fe, Ni or Zn, have been devised and further used to engineer multifunctional and adhesive materials (Figure 5).^83−85^ Interestingly, in our study, we observed that the sizes of the lignin (Kraft lignin) AgNPs decreased with increasing amounts of Ag (Figure 5b,c).^83^ It has previously been suggested that the use of [Ag(NH_3_)_2_]^+^ instead of Ag^+^ will lead to a shift in redox potential toward a lower value, thus leading to a slower reaction (the reduction of silver ions by lignin into AgNPs) and the formation of smaller-sized AgNPs.^79^ Moreover, the higher pH of the [Ag(NH_3_)_2_]^+^ solution will lead to the ionization of the lignin into negatively charged moieties that promote the electrostatic stabilization of Ag. The higher lignin concentration will also enhance the reduction of Ag^+^ into corresponding AgNPs, which in turn leads to a higher rate of reduction and, therefore, faster particle growth.^86^ Hence, a higher amount of silver corresponds to a higher amount of [Ag(NH_3_)_2_]^+^ (higher pH) and, therefore, lower overall lignin concentration relative to silver, which leads to a slower reduction reaction and the generation of smaller-sized AgNPs (Figure 5d).^83,87^

## 3 Combined Catalysis: An Innovative and Sustainable Tool for Engineering Biomaterials

**Figure 6:**
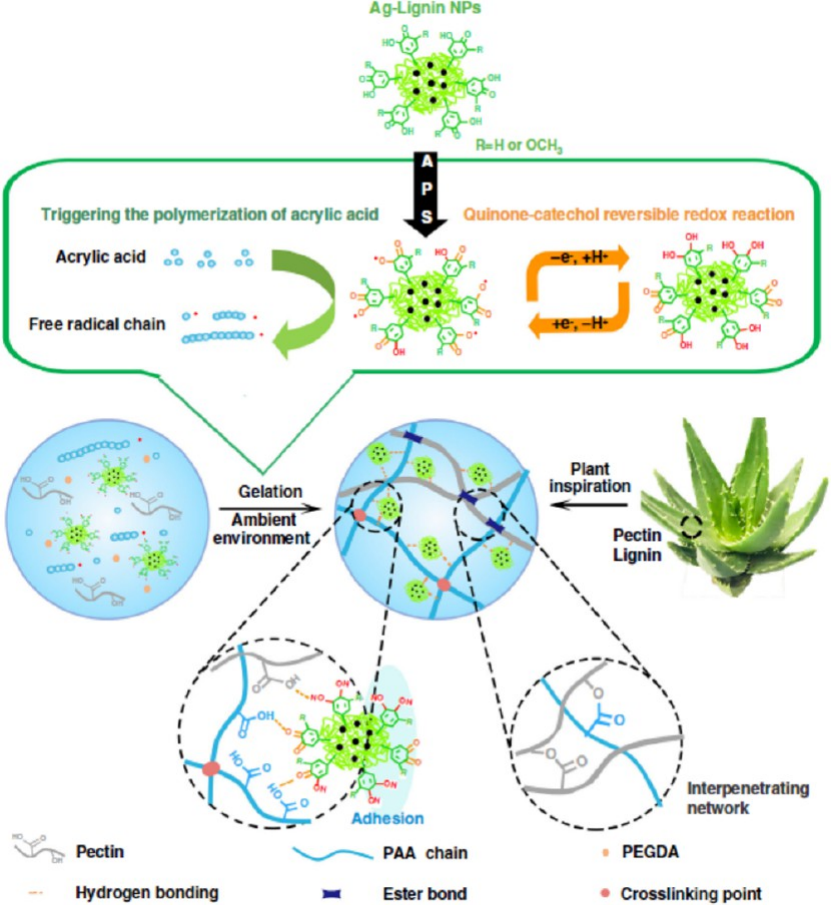
Scheme demonstrating the catalytic strategy for the design of the multifunctional, tough, adhesive, and antibacterial lignin-based hydrogel, its mechanism, and the various interactions within the hydrogel leading to its superior properties. Reproduced with permission from ref (85). Copyright 2019, Gan et al. Licensed under Creative Commons Attribution License 4.0 (CC BY).

**Figure 7:**
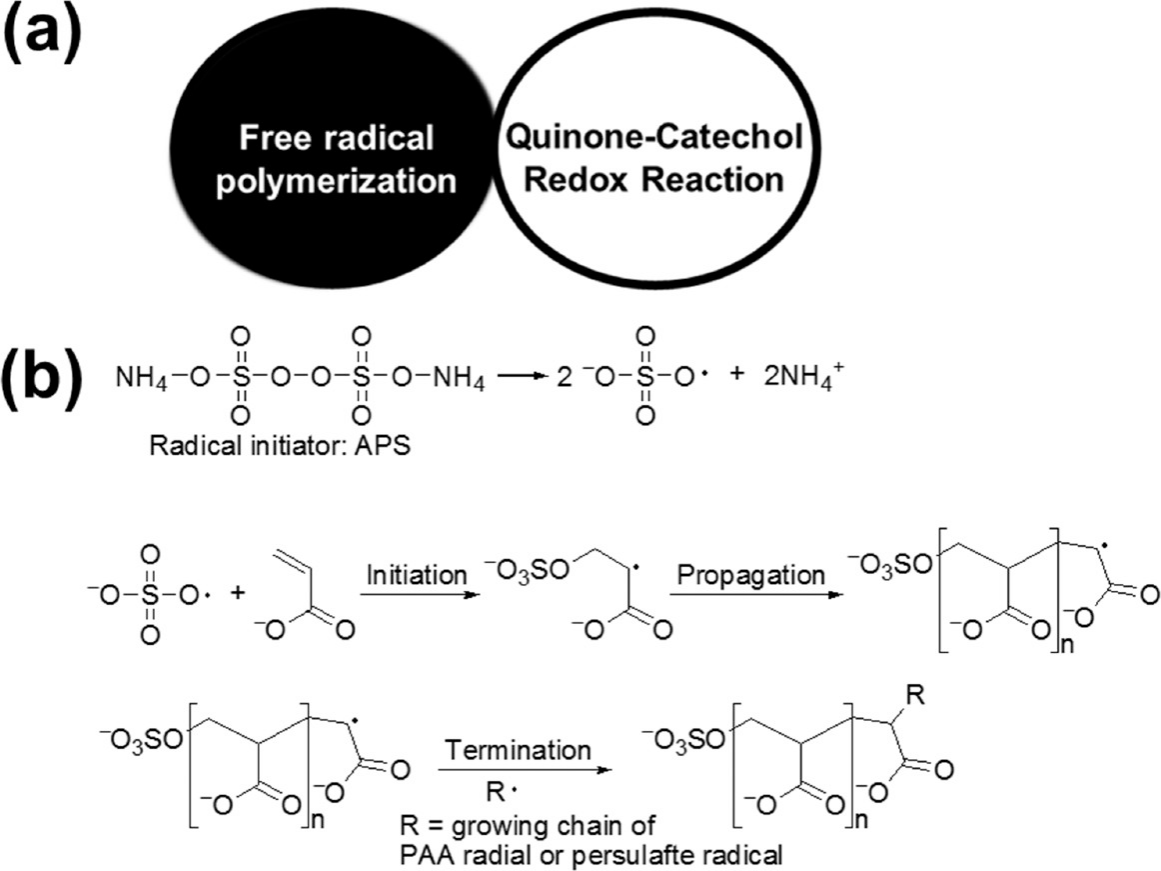
(a) The dual catalytic strategy comprising the combination of the catalytic cycles of silver-catalyzed free radical polymerization and quinone–catechol redox reaction. (b) Scheme demonstrating the mechanism of the free radical polymerization of acrylic acid into poly(acrylic acid) in the presence of the radical initiator APS and the Ag catalyst (not shown in the scheme).

The concept of combined catalysis as mentioned *vide supra* refers to the combination or merging of two or more catalytic cycles or activation modes to promote chemical reactions or the formation of chemical bonds that are not achievable with only one of the catalytic cycles.^5,88^ When carefully designed, this strategy would allow the two individual catalytic cycles to promote each other and synergistically trigger innovative reactions, activation modes, or materials properties (as we will see with some examples *vide infra*) where the single catalyst or catalytic cycle fails.^6^ In the context of combined catalysis for engineering multifunctional lignin-based biomaterials, Gan et al. disclosed the use of cooperative dual catalysis by using one catalyst comprising the two intertwined catalytic cycles of the quinone–catechol redox reaction and free radical polymerization (Figures 1a and 6).^85,89^ The authors introduced the use of lignin (Kraft lignin) AgNPs to trigger a dynamic redox catechol chemistry in the presence of the radical generator ammonium persulfate (APS) to generate radicals and simultaneously promote the polymerization of acrylate substrates (e.g., acrylic acid) in combination with the polysaccharide pectin generating a multifunctional adhesive and tough hydrogel (Figure 6). The hydrogel was further successfully used to promote the healing of infected skin. In the chemical reaction, the Ag catalyst catalyzes both the free radical polymerization^90,91^ and the reversible quinone–catechol redox reaction (Figures 6 and 7a).^92,93^ During the quinone–catechol reversible redox reaction in the oxidation step (generating the radical species), five protons (H^+^) and five electrons (e^–^) are released to provide the phenolic radical species (Figure 6). The toughness and adhesiveness of the hydrogel are a result of the combination of pectin- and poly(acrylic acid)-promoting interpenetrated network and the Ag catalyst with lignin that provides a continuous redox environment.^85^ The presented strategy provides a facile pathway for the polymerization of various acrylate-based substrates and, at the same time, induces the adhesive characteristics from the lignin moiety. The reversible quinone–catechol redox reaction is a well-known reaction that endures in the cells of our body,^94^ and its mechanism proceeds through a proton-coupled electron transfer reaction (Figure 6).^95^ The mechanism of the free radical polymerization reaction as presented in Figure 7b converts the monomer acrylic acid into poly(acrylic acid) in the regenerative presence of APS and an Ag catalyst.^96,97^

**Figure 8:**
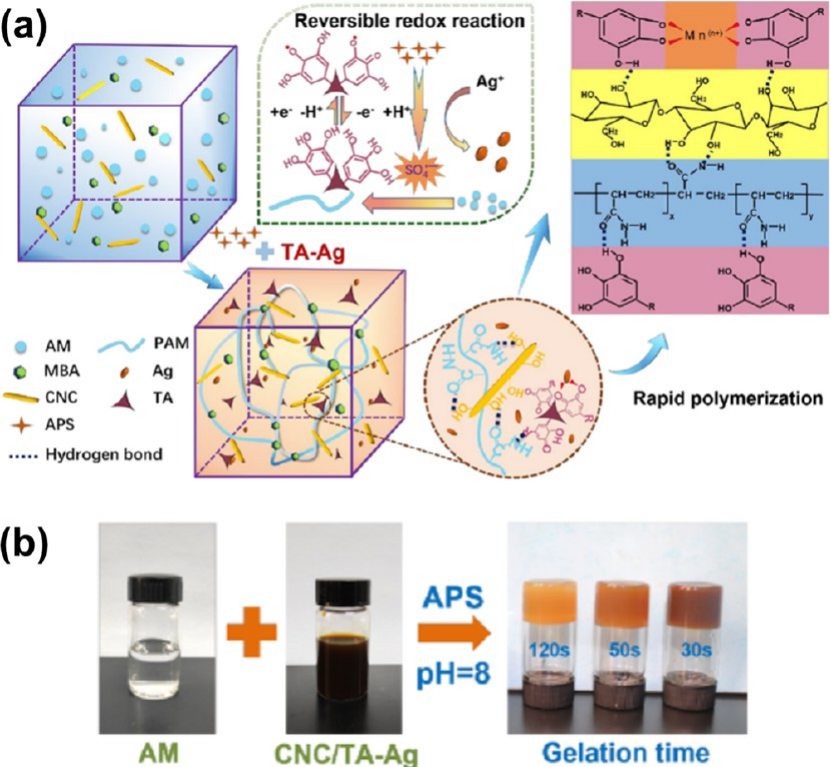
(a) Scheme demonstrating the mechanism of the TA–Ag dual catalysis strategy that promotes fast polymerization of the hydrogel and the various interactions mechanism to provide the hydrogel with its toughness and multifunctional features. (b) Photos demonstrating the fast polymerization of the hydrogels. Reproduced with permission from ref (98). Copyright 2020, American Chemical Society.

A similar strategy was demonstrated by Hao et al. in their tannic acid (TA)–silver dual catalysis approach for the fast polymerization (within 30 s) of acrylamide in the presence of cellulose nanocrystals (CNC)/TA–Ag NPs, cross-linker *N*,*N*′-methylene bis(acrylamide) (MBA), and APS.^98^ In this example, they employed the polyphenolic compound TA containing an abundance of the vital pyrogallol groups instead of lignin (Figures 1a and 8).^99^ It is important to highlight that in Figure 8a, which demonstrates the quinone–catechol reversible redox reaction, it should be clarified that during the oxidation process (providing the formation of the phenolic radical species), 3H^+^ and 3e^–^ are generated, while the formation of the catechol species occurs through a reduction process that traps 3H^+^ and 3e^–^, thereby generating the final catechol species. Besides its multifunctionality, including being stretchable, adhesive, and tough, the hydrogel also demonstrated conductive properties because of the catechol chemistry and the presence of Ag^+^. The authors observed that the ionic conductivity increased by increasing Ag^+^ concentrations.^98^

**Figure 9:**
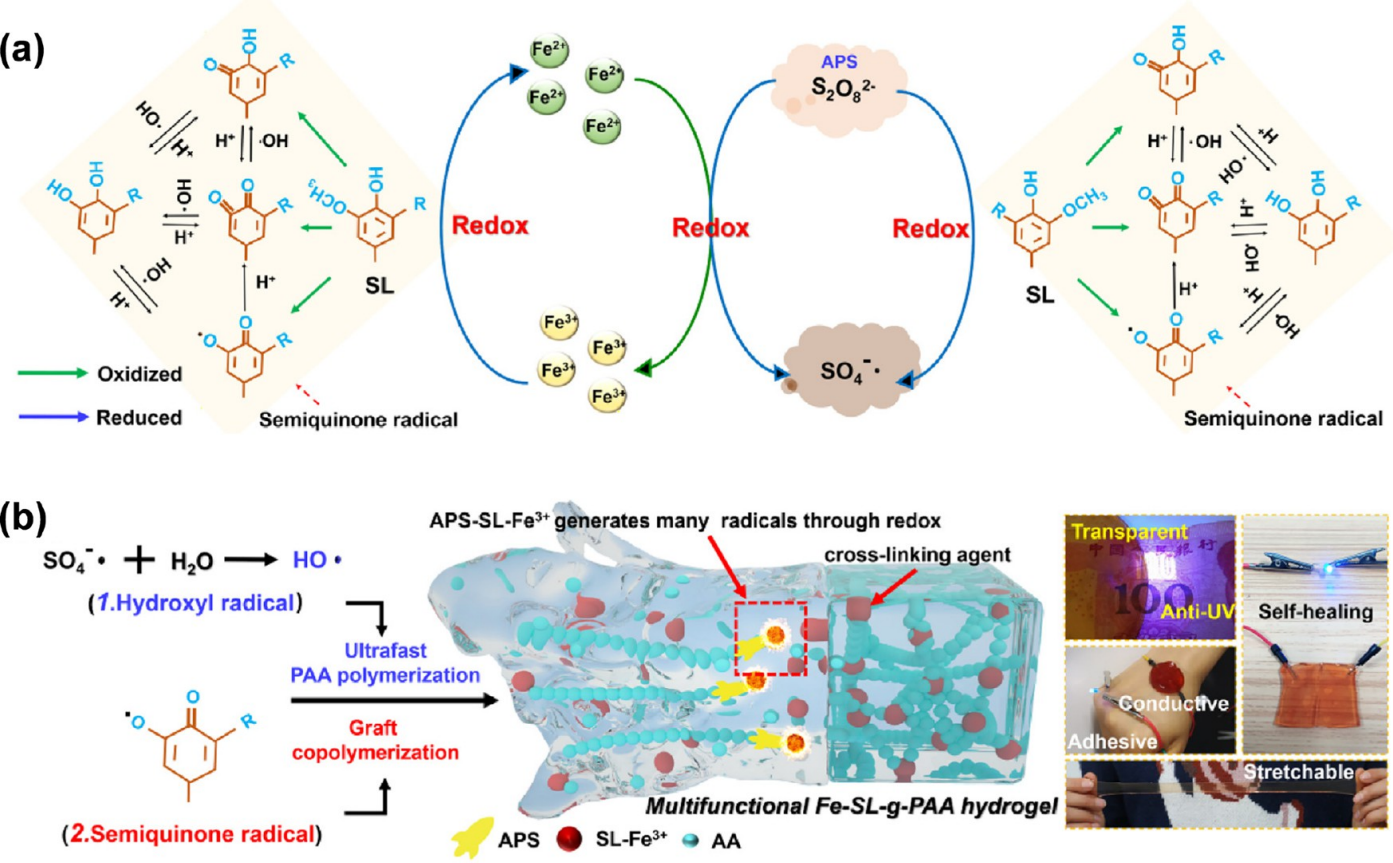
(a) Scheme demonstrating the mechanism of the sulfonated lignin (SL) NPs-Fe^3+^/Fe^2+^ dual-catalyzed reversible catechol chemical strategy and then further (b) free radical polymerization to generate the multifunctional hydrogel. Reproduced with permission from ref (105). Copyright 2020, Elsevier.

Additional reports have disclosed the use of other metals instead of silver, such as ferric ions (Fe^3+^),^100−102^ copper ions (Cu^2+^),^103^ and zinc ions (Zn^2+^),^104^ to engineer multifunctional lignin-based hydrogels. Wang et al. employed sulfonated lignin (SL) NPs-Fe^3+^ to trigger the catechol reversible chemistry and then further rapid free radical polymerization of acrylic acid into a multifunctional Fe-SL-*g*-PAA hydrogel (Figures 1a and 9).^105^ The dynamic oxidation and reduction processes are catalyzed by the iron cations (Fe^3+^/Fe^2+^) in the presence of the APS (Figure 9a). Sun et al. employed lignin (lignosulfonate)-Cu^2+^ for triggering catechol chemistry and further free radical polymerization of hydroxyethyl acrylamide in water and glycerol for the rapid engineering (less than 30 s) of multifunctional organohydrogels with UV-blocking, antifreezing, and antidrying properties.^103^ Moreover, Fu et al. used Zn^2+^ to devise multifunctional hydrogels; however, they used tannic acid as the polyphenolic material.^106^ Jiang et al. used calcium ions (Ca^2+^) and a binary solvent system (glycerol/water) to create a multifunctional organohydrogel with antifreezing and self-healing properties.^107^ Several studies have employed the catalytic ferric–phenolic dynamic redox system for engineering multifunctional hydrogels in combination with the addition of fillers, such as silica nanoparticles^108^ or lithium chloride (LiCl), into the system. These fillers promote the mechanical stability of the nanocomposite hydrogels by endorsing physical cross-linking and the addition of inorganic electrolytes, thereby further adding antifreezing properties.^109^ Mondal et al. used aluminum ions (Al^3+^/Al^2+^) to generate and promote the hydroquinone/quinone redox dynamic process and then further free radical polymerization to generate the LS-PAA-Al hydrogel.^110^

**Figure 10:**
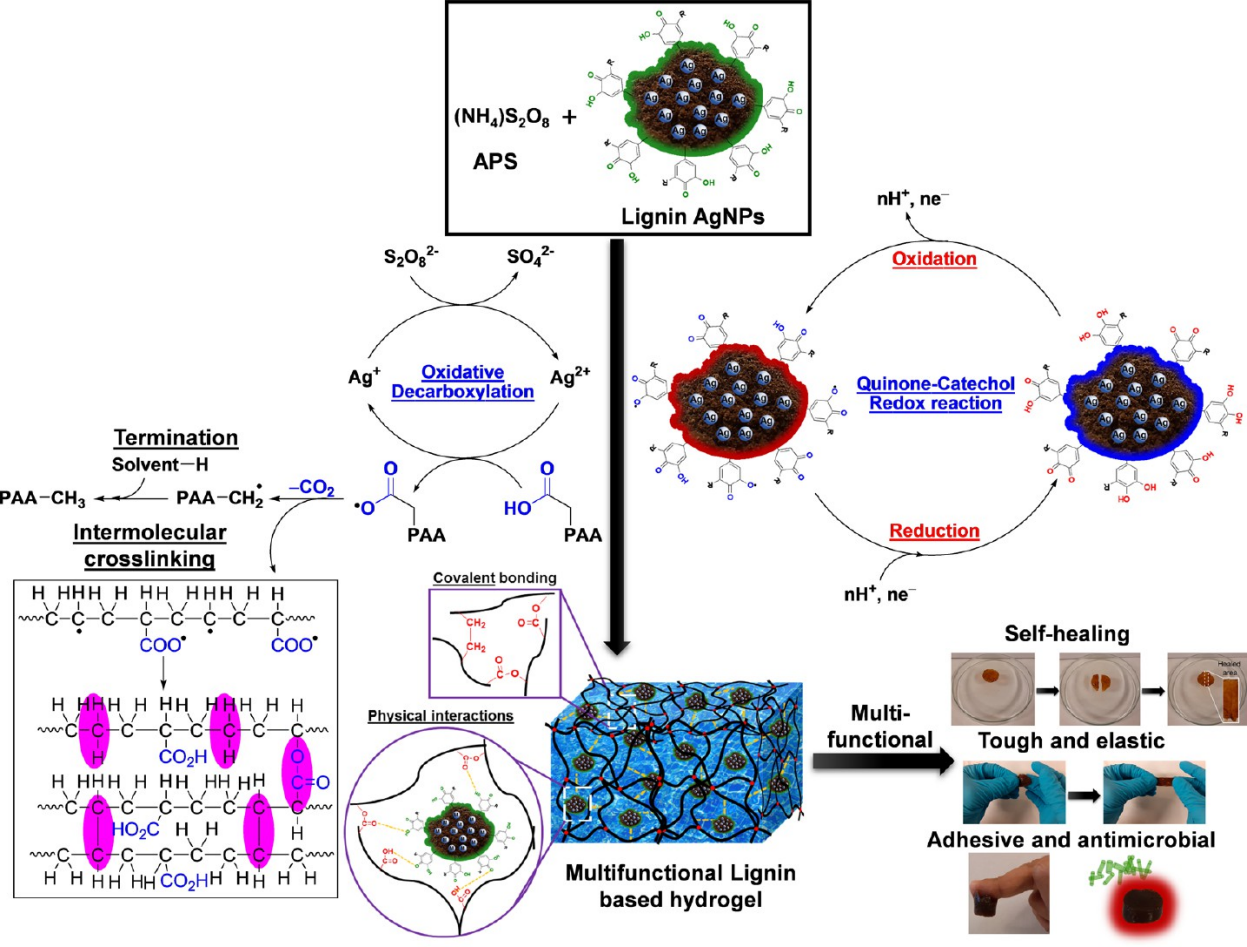
Figure demonstrating the combined catalytic strategy comprising silver-catalyzed oxidative decarboxylation intermolecular cross-linking merged with silver-catalyzed catechol chemistry to generate a multifunctional lignin-based hydrogel with multifaceted characteristics, such as self-healing, adhesive, antimicrobial, tough, and elastic properties. Reproduced with permission from ref (83). Copyright 2020, American Chemical Society.

Furthermore, we disclosed the strategy of combined catalysis for the engineering of a lignin-based multifunctional adhesive, tough, self-healing, and antibacterial hydrogel.^83^ The catalytic strategy encompasses a Ag-catalyzed oxidative decarboxylation intermolecular cross-linking reaction merged with a Ag-catalyzed quinone–catechol redox reaction (Figure 10). In this context, the direct cross-linking of bulk polymers is challenging and generally requires some kind of modifications of the backbone of the polymers or the addition of cross-linking agents.^111^ The presented strategy provides a facile pathway to the catalytic intermolecular cross-linking of bulk materials and polymers postpolymerization without the need for extra modification steps or the use of cross-linking agents, and at the same time, the adhesive and self-healing characteristics are promoted from the lignin moiety. Interestingly, we also demonstrated that without the dual catalytic strategy where the two intertwined catalytic cycles (Ag-catalyzed oxidative decarboxylation reaction and quinone–catechol redox reaction) are combined, the respective single catalytic cycle did not provide the desired hydrogel and its multifaceted characteristics. Nevertheless, synergistic cooperation between the two cycles is essential to provide innovative activation modes and material properties.^83^ For instance, in the absence of a redox environment (the activation of the quinone–catechol redox reaction), only a cross-linked hydrogel was generated without any adhesive property. Conversely, no cross-linked and self-standing hydrogels are observed in the absence of sufficient radicals and the regenerative Ag catalyst that promotes the oxidative decarboxylation reaction. This demonstrates the power of the combined synergistic catalytic system. We also expanded the scope of the catalytic strategy by investigating a wide range of other lignin (Kraft lignin)-based metallic NPs to trigger the quinone–catechol redox reaction and generate adhesive hydrogels (Figure 5).^83^

The strategy of using lignin (Kraft lignin, lignosulfonate, or hydrolyzed lignin) AgNPs to trigger a quinone–catechol redox reaction^112^ followed by cross-linking to provide multifunctional hydrogels has been further expanded by several research groups.^113−119^ Lu and co-workers combined the Ag-catalyzed strategy of an oxidative decarboxylation of citric acid and poly(acrylamide-*co*-acrylic acid), a free radical polymerization of acrylic acid, and a quinone–catechol redox reaction to engineer a versatile hydrogel.^120^ Besides the multifunctionality of the hydrogel, it was also injectable through a needle and fabricated by electrospinning to create micro/nanofibers.

### 3.1 Self-Healing Characteristics

**Figure 11:**
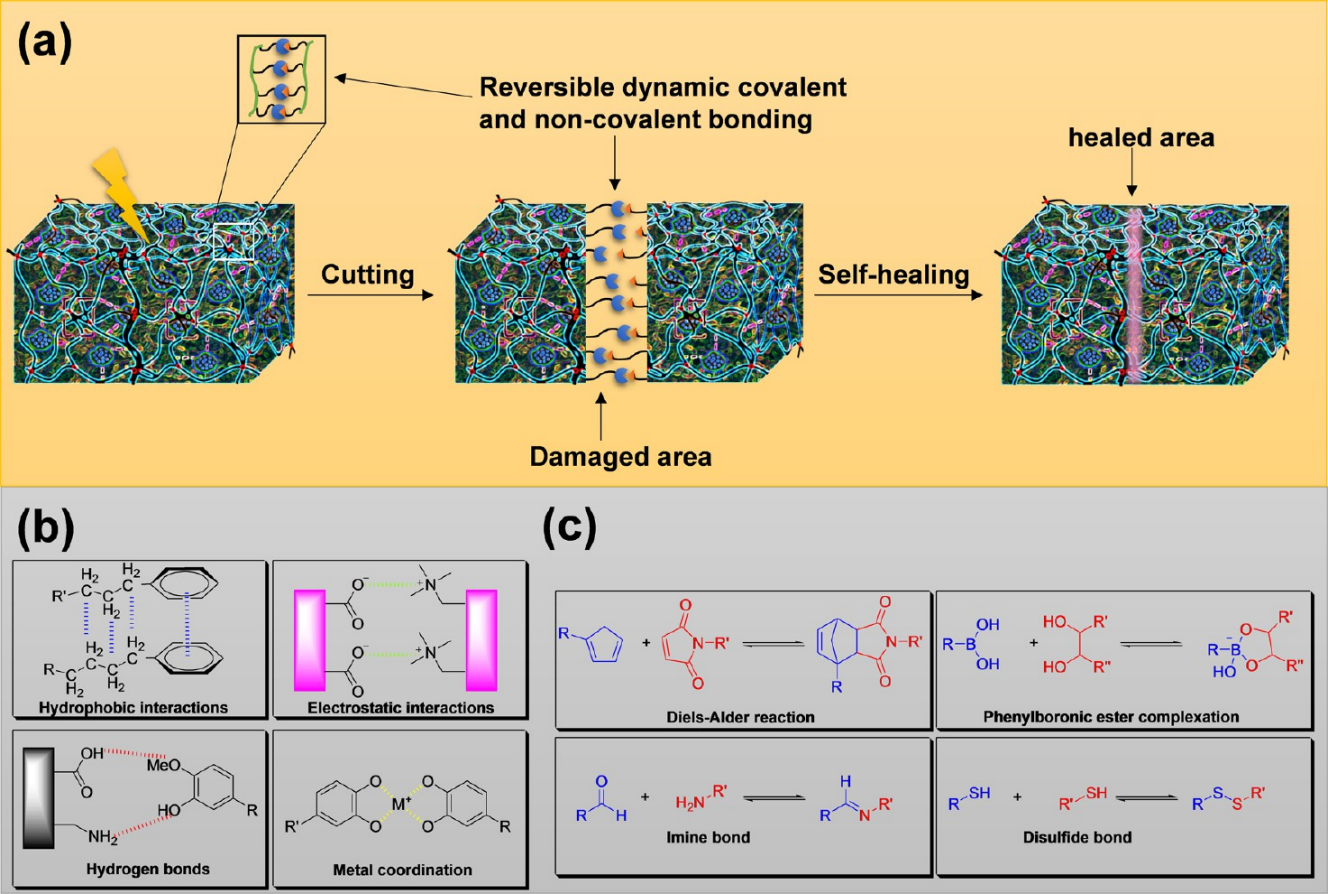
(a) Figure demonstrating the self-healing mechanism of a gel after the gel is damaged by cutting. The gel automatically heals itself through reversible dynamic covalent and noncovalent bonds. (b) Examples of common dynamic noncovalent cross-linking chemistries of hydrogels that promote a self-healing ability. (c) Examples of common dynamic covalent cross-linking chemistries of hydrogels that promote self-healing.

Various biological systems and materials in nature can heal themselves, thus promoting the spontaneous repair of damage and increasing durability and resistance.^121,122^ Lignin can be used as a template in this context for the design of bioinspired self-healing materials. Lignin, with its multifaceted interaction opportunities as mentioned above (Figure 4), provides lignin-based materials with the potential of being self-healing.^123^ Self-healing materials based on lignin that are engineered through catalytic processes would lead to a more sustainable and durable material that is able to repair its damage without any external intervention, which would prolong life and reduce economic loss.^124^ Additionally, lignin-based materials engineered through combined catalysis also add a component to the gel system that promote the self-healing properties derived from the metal catalyst, which provides additional coordination chemistry to the system.^125^ Generally, the design of a material with self-healing property requires the right balance between irreversible (covalent bonds) and reversible (physical bonds) cross-linking within a polymeric system, where the irreversible covalent bonds endorse the structural integrity of the material, and the reversible dynamic noncovalent bonds mainly endorse the self-healing property, respectively (Figure 11a).^124,126^ Common chemical strategies in the design of self-healing hydrogels with noncovalent bonds comprise hydrophobic interactions, electrostatic interactions, hydrogen bonds, and metal coordination (Figure 11b). However, there are also some strategies using covalent cross-linking chemistries that provide dynamic covalent bonds or vitrimers,^127^ which promote permanently cross-linked polymer networks, such as imine bonds (Schiff base),^128^ disulfide bonds,^129^ Diels–Alder reactions,^130^ and phenylboronic ester complexations,^131^ to generate hydrogels with a self-healing ability (Figure 11c).^132^ An additional feature impacting the self-healing ability of a material is the flowability, which promotes the mobile phase to fill the cracked or damaged area and, thus, encourage the healing process.^44^

**Figure 12:**
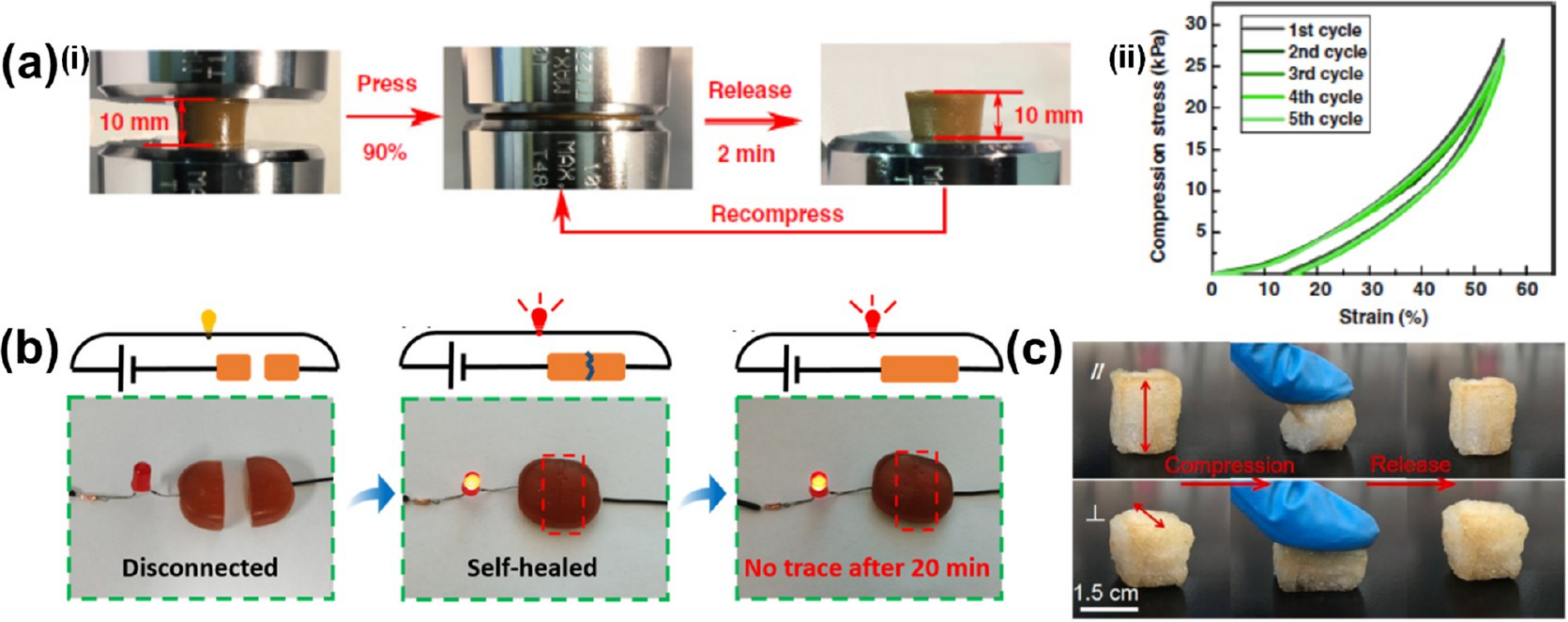
a) (i) Demonstrations of the self-healing features of a gel through compression test and (ii) the corresponding tensile loading–unloading curves. Reproduced with permission from ref (85). Copyright 2019, Gan et al. Licensed under Creative Commons Attribution License 4.0 (CC BY). (b) Image demonstrating the conductivity of a self-healed hydrogel. Reproduced with permission from ref (114). Copyright 2021, Elsevier. (c) An all-wood hydrogel self-healed after compression. Reproduced with permission from ref (101). Copyright 2022, Elsevier.

The application potential of self-healing materials is beyond our imagination. They could potentially be used as drug delivery vehicles (with the ability to prevent any critical damages, such as burst release of a drug reservoir) and coatings (preventing damage to implants by fast recovery or reduction of gel fragmentation and integration of the ruptured gel), among other possibilities. Several reports have demonstrated the self-healing ability of their devised lignin-based materials (Figure 12); however, to our knowledge, no reports to date have demonstrated the importance of the self-healing features of a gel by demonstrating its implementation to any application or solving any challenges.

### 3.2 Universal Adhesion Features: A Bioinspired Approach

**Figure 13:**
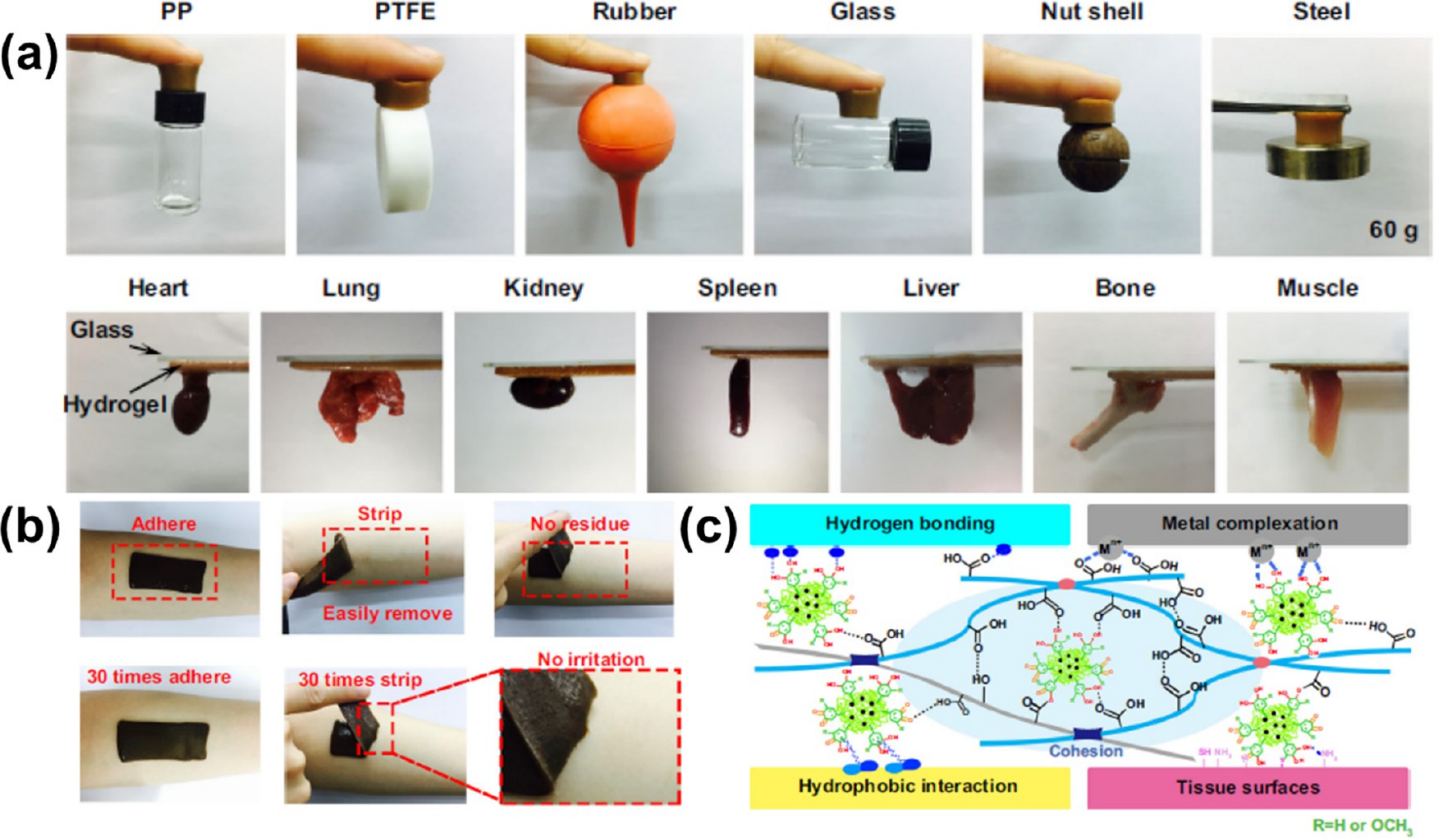
(a) Figure demonstrating the adhesive properties of a lignin-based hydrogel to a wide range of surfaces and materials. (b) Figure demonstrating adhesion of the hydrogel on the skin of the human arm without leaving any residue or causing irritation. (c) Figure demonstrating the adhesion mechanism of the lignin-based hydrogel. Reproduced with permission from ref (85) Copyright 2019, Gan et al. Licensed under Creative Commons Attribution License 4.0 (CC BY).

Engineering materials that integrate decently or adhere to other materials or surfaces, such as metals, woods, papers, glass, plastics, ceramics, and tissues, are important to ensure good integration, form a strong barrier, and stay intact within the material or surface.^133^ Adhesion to various surfaces and materials proceeds through the formation of chemical or physical interactions between the two entities.^134^ However, the engineering of materials with adhesion ability to a wide range of materials and surfaces under various conditions, such as dry and wet environments, cold and hot environments, and hard and soft surfaces, is a daunting challenge.^49,135,136^ Bioinspired strategies mimicking the chemistry of the sticky materials generated by creatures in nature, such as mussels (they have demonstrated universal adhesive ability derived from their distinct protein-rich secrete containing dopamine and catechol groups), could potentially address the challenge and promote the design of universal adhesive materials.^75,137,138^ In fact, these types of engineered materials have emerged in biological and biomedical science where they have demonstrated strong adhesion to biological tissues with broad applications, such as tissue repair,^139^ tissue sealants,^140−142^ hemostatic materials,^143^ drug delivery,^144^ flexible electronics,^145^ and wound dressing.^146^ Lignin with its exceptional structure has been demonstrated to mimic the chemistry of the mussel’s secrete, which promotes a wide range of interaction possibilities and, thus, adhesion to a wide range of substrates and surfaces (Figure 13).^83^ Most of the reports highlighted in this review have demonstrated the universal adhesion ability of their engineered lignin-based materials to a wide range of surfaces and materials without requiring any chemical modification or pretreatment of the biomaterial for the adhesion to proceed (Figure 13a).^83,85^ One interesting example within this framework is the lignin-based organohydrogel that was engineered with adhesive properties to a wide range of surfaces and materials both at room temperature and even at subzero environments.^103^ Nevertheless, it would be interesting to further investigate and understand the long-term performance of the organohydrogel’s adhesion property in subzero environments.

### 3.3 Dual Antimicrobial Actions

**Figure 14:**
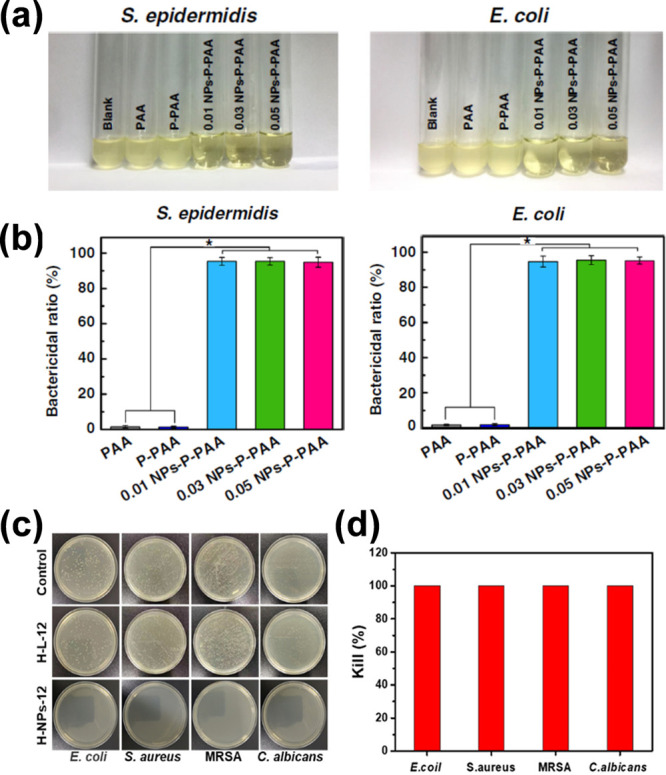
(a) Photos of *Staphylococcus epidermidis* and *Escherichia coli* solution cocultured with the Ag-lignin NPs-based hydrogels after 1 day and (b) the bacterial ratio of the hydrogels. Reproduced with permission from ref (85). Copyright 2019, Gan et al. Licensed under Creative Commons Attribution License 4.0 (CC BY). (c) Photos of the bacterial colonies using *E. coli*, *Staphylococcus aureus*, methicillin-resistant *S. aureus* (MRSA), and *Candida albicans* on agar plates incubated for 2 h with the surface of lignin-based hydrogel, and (d) the surface antibacterial properties of the hydrogels. Adapted with permission from ref (115). Copyright 2021, Deng et al. American Chemical Society.

To date, scientists around the world are trying to innovate and create better solutions to battle the current challenges associated with the multidrug resistance of pathogenic microorganisms^147^ and their impact on the healthcare system and infectious diseases.^148−151^ In light of this, nature-based materials are generally considered safe^152^ and renewable and have demonstrated additional properties, such as antioxidant behavior,^153^ a broad antimicrobial spectrum,^154^ activity against sensitive and resistance pathogens, and some have been shown to reverse antibiotic resistance.^155^ Therefore, they could be a good candidate to address the abovementioned challenge and provide alternative or complementary technologies to conventional antibiotics.^156−158^ Several reports have demonstrated that polyphenols, such as lignin,^69,159^ possess a broad spectrum of antimicrobial activities (Figure 14).^45,160^ Generally, the antimicrobial activity of industrial lignin is weak^69,161^ and needs either further processing^162^ or modification to boost this property.^163−165^ In this context, catalysis or combined catalysis can promote the enhancement of the antimicrobial activity of lignin-based materials through activation of the lignin moiety,^83,85^ selective modification,^166−168^ or selective depolymerization of lignin-type polyphenolic structures.^169,170^ Moreover, because of the complex polyaromatic structure of lignin, selective catalysis^171,171−176^ is vital to generate the desired structure for further tailoring into an adhesive,^177−179^ drug delivery matrix,^180,181^ antimicrobial materials,^167^ composite,^182,183^ catalyst,^184,185^ and sensor.^31,107,186^ This would also further promote the valorization of lignin into high-value products.^187,188^

**Figure 15:**
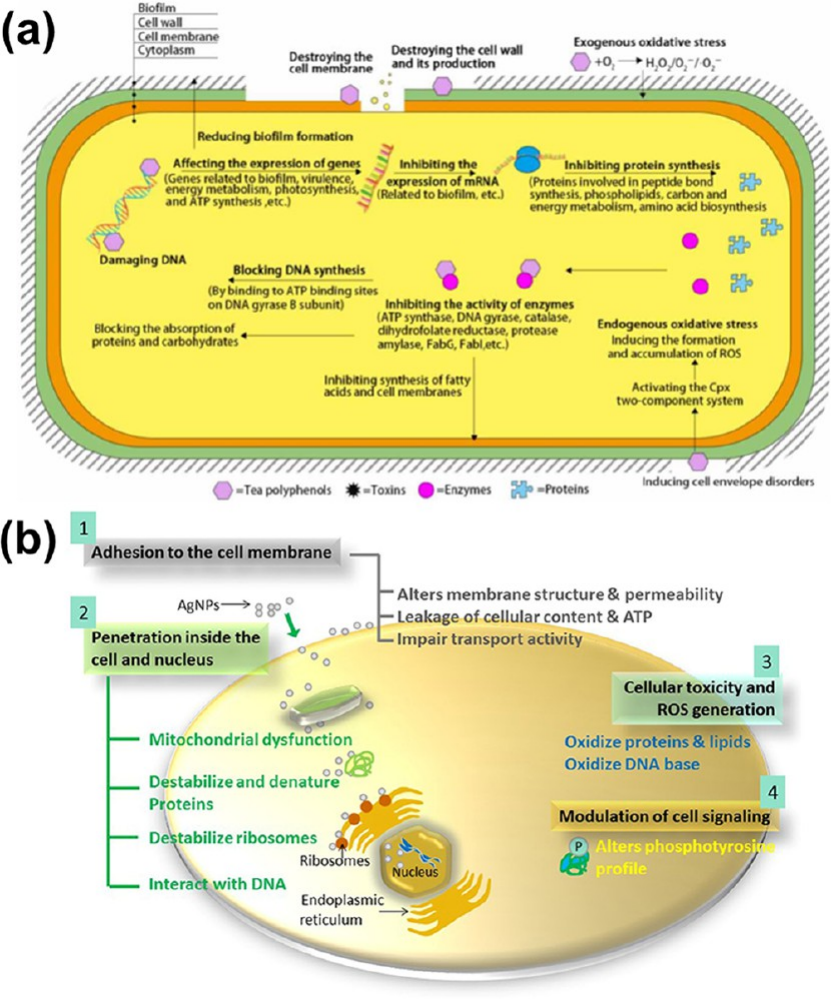
(a) Figure demonstrating the antimicrobial mechanism of polyphenols. Reproduced with permission from ref (191). Copyright 2022, Li et al. Licensed under Creative Commons Attribution License 4.0 (CC BY). (b) Figure demonstrating the antimicrobial mechanism of AgNPs. Reproduced with permission from ref (197). Copyright 2016, Dakal et al. Licensed under Creative Commons Attribution License (CC BY).

The antimicrobial mechanism of action of polyphenols is believed to proceed via several pathways, such as interactions with the cell membrane and cytoplasm, thereby leading to damage of the bacterial membrane,^167^ enzyme inhibition, and suppression of bacterial biofilm formation (Figure 15a).^189−191^ Most of the lignin-based multifunctional materials presented in this review with antibacterial activities proceed through dual mechanistic actions derived from the polyphenolic lignin and the metallic element used as the catalyst to promote the cross-linking or polymerization process (Figure 14).^83,85,110,114,115,118^ Elements (metal nanoparticles or metal ions) such as AgNPs are known to have antimicrobial activity, and their mechanism or mode of action is well known.^192^ For instance, the AgNPs or their respective leached Ag^+^ promote antimicrobial activity through various approaches, such as by interaction with the cell wall, which leads to leakage; by inhibition of protein synthesis, which encourages DNA damage; and by degradation through the promotion of reactive oxygen species (ROS) activation and interaction with numerous metabolic mechanisms (Figure 15 b).^193−197^

## 4 Conclusion and Future Direction

The smooth transition of the paradigm shifts our society is undergoing toward a more sustainable world and lifestyle requires the advancement of innovations in green chemistries and their respective applications for the engineering of sustainable materials. There is no doubt that multifunctional sustainable materials will play a major role in promoting these changes. We envision one material that is durable, sustainable, and with the ability to demonstrate a wide range of characteristics and properties that can solve numerous challenges. Lignin, with its abundance and distinct features, could play a major role as a template for engineering multifunctional materials with the ability to solve various challenges in a multifaceted approach. For instance, engineering a material that is robust and tough, antimicrobial, self-healing, adhesive, conductive, UV-resistant, and environmentally adaptable all in one would have a great impact on its application. Lignin possesses several characteristics that make it a desirable and distinct material: (1) it has an exceptional structure, (2) it is abundant, (3) it is sustainable, and (4) it contains functional groups that are easily modified. Moreover, despite the many studies presented in this review employing combined catalysis for the engineering of multifunctional lignin-based materials, the materials’ multifunctionality is not harnessed to the fullest to solve multiple challenges simultaneously with one material. We have shown one example where a multifunctional material is used for the healing of infected wounds utilizing the ability of the material to simultaneously promote wound repair and employ its antibacterial ability. Hence, to further expand and completely explore the multifaceted features of the materials into important applications to solve great challenges would further demonstrate the power and importance of these materials. For instance, the self-healing ability of the materials could be used to create robust and sustainable fabrics and coatings able to restore themselves upon damage or fracture and at the same time prevent any potential future infection or biofilm formation. Further expansion and exploration of lignin’s catalytic performance in engineering multifunctional sustainable materials could be further advanced by the in-depth evaluation of the behavior and performance of lignin obtained from the various extraction processing approaches and lignin sources. Furthermore, we have observed several examples in this review that illustrate the use of cellulose materials merged with lignin either as lignocellulose or cellulose lignin composite through combined catalysis. Cellulose is an abundant, versatile, and sustainable material and has great potential to further expand the field of combined catalysis for engineering multifunctional lignin-based sustainable materials by adding its distinct structure (which promotes hydrogen bonds) and functionalities (an abundance of hydroxyl groups that allow for selective catalytic modification to tailoring its physical and chemical properties). Hence, selective catalytic modification of cellulose through the various described combined catalytic system would further promote and expand the future research direction of the discussed topic by merging these two important sustainable materials (lignin and cellulose). For instance, through the selective catalytic modification of cellulose, the promotion and allowance for various agents, such as metal ions, to bind could further be used for the catalytic activation of lignin to promote the catalytic quinone–catechol redox reaction and, thus, merge activated cellulose and lignin into a multifunctional sustainable material. We hope that this review will stimulate the scientific community and material scientists to invent chemical approaches and material properties by using the concept of combined catalysis and to take some inspiration from the field of organic synthesis. Therefore, future directions are anticipated to explore other combined catalytic cycles and reactions besides the catalytic quinone–catechol redox reaction combined with either free radical polymerization or oxidative decarboxylation reactions.

## VOCABULARY

catalysis: a process where the speed of a reaction is promoted by using a substance named catalyst, which is not consumed during the reaction.
valorization: the process of converting low-value products into high-value products.
organohydrogel: a class of 3D gels comprising a cross-linked polymeric network that can swell in liquid organic and aqueous media.
oxidative decarboxylation reaction: a reaction where carbon dioxide (CO_2_) is removed from a carboxylic acid moiety through an oxidation process.
free radical polymerization: a polymerization method used for the formation of cross-linked polymers using free radicals created in chemical reactions.
green chemistry: the design of chemical processes and products with the aim of reducing or eliminating the use or generation of hazardous substances.
nanoparticle: a small particle with sizes between 1–100 nm.
